# Transcriptome and phytohormone changes associated with ethylene-induced onion bulb dormancy

**DOI:** 10.1016/j.postharvbio.2020.111267

**Published:** 2020-10

**Authors:** M. Carmen Alamar, Maria Anastasiadi, Rosa Lopez-Cobollo, Mark H. Bennett, Andrew J. Thompson, Colin G.N. Turnbull, Fady Mohareb, Leon A. Terry

**Affiliations:** a Cranfield University, Bedfordshire, MK43 0AL, UK; b Department of Life Sciences, Imperial College London, London, SW7 2AZ, UK

**Keywords:** *Allium cepa*, Sprout elongation, Ethylene supplementation, Abscisic acid, Cytokinin, Gene expression

## Abstract

Control of dormancy and sprouting in onion bulbs is commercially important for postharvest management. Although ethylene application is sometimes used to extend dormancy, the underlying mechanisms regulating dormancy transition remain unclear. Since the sprout leaves emerge from the bulb baseplate, we used this tissue to assess the impact of ethylene treatment and storage time on the hormone profile and the transcriptome. Reads from 30 libraries were assembled and annotated, with 94,840 unigenes retained after filtering. The *de novo* transcriptome assembly was of high quality and continuity (N50: 1809 bp, GC content: 36.21 %), and was used to analyse differential expression and Gene Onotologies. Across two years, applied ethylene resulted in delayed dormancy break and reduced post-dormancy sprout vigour. Ethylene supplementation enhanced endogenous ethylene production and caused a transient climacteric-like increase in respiration. Significant changes in hormone and associated transcript profiles occurred through storage and in response to ethylene. In particular, abscisic acid (ABA) and its metabolite phaseic acid (PA) increased under ethylene during the longer dormancy period; however, cytokinin increases observed during storage appeared largely independent of ethylene treatment. Several hormone-related transcripts showed differential expression over time and/or in response to ethylene. Expression of ethylene biosynthesis (*ACO*), receptor (*EIN4*) and transcription factor (*EIL3*) genes were modified by ethylene, as were ABA biosynthesis genes such *NCED,* and cytokinin biosynthesis genes such as *LOG* and *CKX*. We conclude that ethylene substantially modifies expression of genes in several phytohormone pathways, and some of these changes may underlie the dormancy-extending effects of exogenous ethylene.

## Highlights

• A *de novo* onion transcriptome was developed to investigate dormancy transition.
• Continuous ethylene induced a climacteric-like respiration peak in stored onions.
• Ethylene upregulated ethylene biosynthesis transcripts, *ACO1*, in onion baseplates.
• The ABA biosynthesis gene *NCED* was upregulated under ethylene supplementation.
• *t*ZRP and IPRP are potential markers of onion dormancy status.

## 1 Introduction

Onion bulbs are typically stored to maintain their supply year-round, whilst early dormancy break greatly reduces quality and marketability (Terry et al., 2011). Dormancy can be extended commercially by application of the herbicidal growth inhibitor maleic hydrazide, or by controlled atmosphere treatment (high carbon dioxide, low oxygen), but an improved understanding of the mechanisms of dormancy may provide a more powerful means to extend storage.

Bulb dormancy can only be understood in the context of the preceeding bulb development and morphology. The true primary stem of an onion plant is a compressed baseplate made up of very short phytomers (leaf node bearing a leaf, axillary bud, and the subtending internode), whereas the pseudostem is formed from the concentric leaf sheaths bearing the leaf blades. Bulb development begins with the swelling of the sheaths bases in the blade-bearing outer leaves; the sheaths of the inner leaves (storage sheaths) also swell, but blade development aborts. The smaller, innermost leaves (sprout leaves) are bladed and grow into sprouts after dormancy is broken. In addition, one or more axillary buds develops inside the bulb, forming up to one third of the mature bulb mass through the swelling of its own outer leaves (Jones and Mann, 1963; Brewster, 2008). As the bulb matures, the initiation of new leaves and expansion of leaves in the mature bulb is arrested, the pseudostem softens and collapses (“fall down”), and the bulb enters a temporary, developmentally-determined endodormant state (Brewster, 2008). Once the endodormancy period is over, the bulb may enter an ecodormant state where non-permissive external environmental factors—usually low temperature—prevent sprouting (Lang et al., 1987). Dormancy break is characterised by elongation of cells in the pre-existing sprout leaves and by mobilisation of nutrients in the storage sheaths (Pak et al., 1995), whilst sprouting is recorded when the sprout leaves extend beyond the neck of the harvested mature bulb (Brewster, 2008).

There are multiple reports describing the cross-talk of different phytohormones associated with dormancy break and sprout leaf elongation (Hartmann et al., 2011; Cools et al., 2011; Chope et al., 2012), as well as with mobilisation of non-structural carbohydrate reserves (Davis et al., 2007), particularly fructans (Valluru, 2015; Ohanenye et al., 2019). In general, sprout elongation is thought to be initiated by the action of promoting hormones, such as CKs and gibberellins whilst being suppressed by the antagonist action of the inhibitory hormone ABA. It has been proposed that ABA is mostly synthesised in the leaf blades, and then translocated to the bulb during the growth period, where it accumulates (Matsubara and Kimura, 1991). Chope et al. (2007b) showed that a decrease in ABA concentration in onion bulbs started just after harvest, during the curing period; this ABA depletion continued during cold storage and minimum levels of ABA coincided with the onset of sprout elongation (Chope et al., 2006, 2007a). Moreover, they suggested that ABA accumulation during bulb growth could be correlated with delayed sprout elongation in-store (Chope et al., 2006).

Exogenous ethylene application has been used to extend dormancy in onion bulbs (Buffler, 2009; Downes et al., 2010; Ohanenye, 2019), and it has long been known that ethylene inhibits elongation of young leaves in bulbous plants (Kamerbeek and De Munk, 1976). Ethylene is considered to be an inhibitor of leaf growth that reacts to both biotic and abiotic stresses. In particular, within the ethylene signalling pathway, ethylene responsive factors (ERFs) are proteins known to inhibit cell division *via* gibberellin pathways, and to slow down cell expansion by reducing the level of the expansins that regulate cell wall extension (Dubois et al., 2018). However, understanding of these pathways comes from studies of leaf growth in model plants, and little is known about the significance of these regulatory pathways in controlling growth of *Allium* sprout leaves during and after dormancy. Cools et al. (2011) used a 60-mer oligonucleotide microarray designed from expressed sequence tag (EST) data representing 13,310 unique onion sequences to investigate the differential expression of onion genes in response to exogenous ethylene and 1-methylcyclopropene (1-MCP, an ethylene antagonist), applied either individually or in combination. They found that continuous ethylene treatment during long-term cold storage reduced onion sprout elongation, whilst upregulating transcripts annotated as *EIN3* transcription factor, along with other transcripts consistent with growth inhibition, *e.g.* related to ethylene biosynthesis (Cools et al., 2011). Although there is not yet an onion genome reference assembly, the onion transcriptome has recently been more fully described in several studies using RNA sequencing (RNA-Seq) and *de novo* transcriptome assembly (Shon et al., 2016; Zhang et al., 2016, 2018; Galsurker et al., 2017, 2018, 2020); this approach now provides greater power to investigate transcriptome changes with superior genome coverage and sensitivity.

In this study, we investigated the mechanism by which ethylene extends onion bulb dormancy by measuring hormone and transcriptome responses. Since the sprout leaf emerges from the bulb baseplate, we targeted analysis to the baseplate tissue to investigate its potential role in controlling the early events during initiation of sprouting.

## 2 Materials and methods

### 2.1 Overview of experimental design

**Fig. 1:**
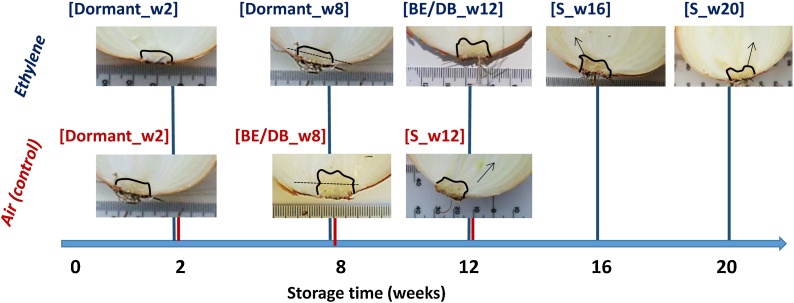
Dormancy break phenotyping of cv. Sherpa bulbs subjected to continuous ethylene supplementation (10 μl l^−1^) and air (control) during cold storage at 1 °C and *ca*. 45 % relative humidity. Baseplate expansion (BE) occurred at week 8 (w8) and week 12 (w12) of cold storage for control and ethylene-treated bulbs, respectively; this point was considered as dormancy break (DB). Storage stopped once all bulbs showed sprout elongation (S). A total of 30 samples (3 replicates per time point) were sent for RNA sequencing: whole baseplates were analysed except from those at week 8 where top and bottom sections (as indicated by a transversal line) were studied.

Bulbs of cv. Sherpa were cold-stored at 1 °C either under air (control) or with a continuous supplementation of air plus ethylene at 10 mg kg^−1^ (Fig. 1). Bulbs were sampled across different developmental stages: dormant, at dormancy break, and during sprout elongation; this was repeated over two seasons. The respiration rate, sprout and root incidence (presence *vs*. absence), and sprout elongation were evaluated throughout cold storage to provide the physiological context. The profiles of ABA, ABA metabolites and CKs were determined in whole onion baseplates (weeks 2, 12, 16 and 20) and in baseplates bisected transversely (week 8). The latter sampling point was selected since control baseplates at that time appeared to have expanded vertically compared to ethylene-treated ones (Fig. 1); based on this phenotyping, week 8 was considered the time to dormancy break in control bulbs. Moreover, RNA-seq data was obtained from exactly the same baseplate tissues sampled for phytohormone measurements, and used to perform a *de novo* transcriptome assembly and to identify differentially expressed genes (DEGs).

#### 2.1.1 Plant material

Postharvest storage trials were performed in two consecutive harvesting seasons (2013–2014) using onion bulbs cv. Sherpa. Bulbs were harvested from a field in Stapleford (sandy clay loam), Newark on Trent, Nottinghamshire, UK; and cured for 3 weeks at 28 °C, followed by 3 weeks at 26 °C. In 2014 (Year 2), ‘Sherpa’ bulbs were also harvested from Nottinghamshire, UK and cured for 4 weeks at 26 °C. Different curing programmes were followed by each grower to accommodate specific climate and bulb conditions at harvest time; however, no major differences (*e.g.* commercial skin finish) were found between seasons (data not shown). Sprout suppressants (*e.g.* maleic hydrazide) were not applied at pre-harvest stages for any of the two harvest seasons.

### 2.2 Experimental design and bulb sampling

On arrival at Cranfield University, the bulbs were transferred to 100-l storage boxes and stored in a cold room at 1 °C. All experiments were arranged in a completely randomised design with three replicates. The year 1 and 2 bulbs were subjected to two treatments: continuous ethylene supplementation at 10 mg kg^−1^ or continuous air as previously described by Cools et al. (2011). Ethylene gas sourced from a 50 l cylinder of ethylene concentration of 5000 μl l^−1^ (BOC, Surrey, UK) was reduced to 10 μl l^−1^ by mixing ethylene with air through a manifold (HNL, Engineering Ltd, Stockton-on-Tees, UK), according to Amoah et al. (2017). The ethylene concentration in the boxes was checked on a weekly basis by withdrawal of gas samples from each of the storage boxes and following the method described by Chope et al. (2007a).

Ten and nine sampling points were used for year 1 and 2, respectively. The first sampling point was conducted on arrival of the bulbs (time 0); afterwards, sampling was performed bi-weekly until sprout length in control samples was *ca*. 40 % in comparison to bulb height. During sampling, the bulbs weights were taken, the respiration rates measured, sprout assessment recorded, and the baseplates excised for biochemical and RNAseq analyses. At each sampling point, three bulbs (biological replicates) were collected in triplicates, per treatment and throughout the storage duration.

### 2.3 Sample preparation

Onion bulbs were cut into half from top to bottom with a sharp stainless knife. The baseplates from both halves were excised and immediately snap-frozen in liquid nitrogen. For year 2, week 8 baseplates were further divided into top and bottom sections (Fig. 1; **Figure S1**); at this point sprout elongation started in control onions while ethylene-treated bulbs did not present sprout elongation, yet shown thicker baseplates. All samples were stored at −80 °C before and after lyophilisation in a freeze-drier (Scanvac, Lynge, Denmark) in the dark for 7 days.

### 2.4 Respiration rate (RR)

Real-time RR measurements were taken at each sampling point using the Sable Respirometry System (Model 1.3.8 Pro, Sable Systems International, NV, USA), in accordance with the method described previously by Alamar et al. (2017). Each replicate (3 replicates x 3 biological replicates [onion bulbs]) were taken out from the storage boxes and placed on the laboratory bench for a minimum of two hours to be acclimatised to room temperature. Each replicate was placed in a 3 l sealed gas jar with gas inlet for air supply and outlet for respiration rate measurement (as CO_2_ production by the onion bulbs). The amount of CO_2_ produced was measured in millilitres per hour (ml h^−1^). The RR values were determined in relation to bulb weights to get the final respiration rate values as mg kg^−1^ h^−1^, as previously described by Cools et al. (2011).

### 2.5 Sprout and root assessment

Sprout and root incidence were recorded as previously described elsewhere (Chope et al., 2007a; Cools et al., 2011). Bulbs were cut in half (vertically from top to bottom) and checked for presence or absence of roots, and sprout elongation. Sprout elongation was considered when sprouts were ≥ 5 mm and the tip was yellowish. Where present, sprout length, and onion bulb height (mm) were recorded; the mean length value for existing sprouts per bulb was used for further calculations. Sprout length was presented as a percentage with respect to the total bulb height. Internal photographs were also taken at each sampling point to record both sprouting and baseplate developmental stage (**Figure S1**). The difference in sprout elongation was assessed by comparing the sprout length of onion bulbs at similar physiological state and under both treatments; for example control bulbs at week 8 (dormancy break) were compared with ethylene-treated bulbs at dormancy break (week 10). Thus, plotting the sprout length against the weeks after dormancy break provides an indication of sprout vigour (cv. Sherpa –year 2 only).

### 2.6 Phytohormone extraction and quantification

Freeze-dried samples (*ca*. 2 mg) were powdered using a TissueLyser (Model II, QIAGEN, Hildend, Germany) for 2 min at 30 Hz, and further extracted and quantified by LC/MS-MS according to Morris et al. (2018). An internal standard mixture containing 1 ng of the ollowing compounds was added at the beginning of the extraction process: [^2^H_5_]-*trans*-zeatin (d5-*t*Z); [^2^H_5_]-*trans*-zeatin riboside (d5-*t*ZR); [^2^H_5_]-*trans*-zeatin riboside-5ˊ-monophosphate sodium salt (d5-*t*ZRP); [^2^H_6_]-N^6^-isopentenyladenine (d6-IP); [^2^H_6_]-N^6^-isopentenyladenosine (d6-IPR), [^2^H_6_]-N^6^-isopentenyladenosine-5ˊ-monophosphate sodium salt (d6-IPRP); (-)-5,8ˊ8ˊ8ˊ-d_4_-abscisic acid (d4-ABA); (-)-7ˊ,7ˊ,7ˊ-d_3_-phaseic acid (d3-PA); (-)-7ˊ,7ˊ,7ˊ-d_3_-dihydrophaseic acid (d3-DPA); (+)-4,5,8ˊ,8ˊ,8ˊ-d_5_-abscisic acid glucose ester (d5-ABA-GE); (±)-5,8ˊ,8ˊ,8ˊ-d_4_-7ˊ-hydroxy-ABA (d4−OH-ABA). Deuterated and non-deuterated ABA metabolites: (-)-DPA, (+)-ABA-GE, (-)-PA, (±)-7ˊ-hydroxy-ABA, were obtained from the National Research Council of Canada-Plant Biotechnology Institute; (±)-ABA was purchased from Sigma-Aldrich; and the rest of the standards were obtained from OlChemIm, Olomouc, Czech Republic. Final phytohormone concentrations were expressed as nmol kg^−1^ per dry weight.

### 2.7 RNA extraction

RNA sequencing was done for 2014 samples only. Total RNA from 30 onion baseplate samples at different developmental stages (dormant, dormancy break and sprout elongation) was isolated using the PureLink RNA Mini kit (Ambion) following manufacturer instructions. Specifically, five (week 2, 8, 12, 16 and 20) and three time points (week 2, 8 and 12) were studied (in triplicate) for ethylene-treated and control bulbs, respectively. For all time points and both treatments, except from those at week 8, RNA was extracted from a pool of three individual baseplates, per replicate. For week 8, baseplates were divided into top and bottom sections (Fig. 1), to investigate spatial differences. Fresh-frozen samples were ground for one minute at 30 Hz with a TissueLyser II (QIAGEN); 1 ml of lysis buffer containing 1 % β-mercaptoethanol was added immediately after, and shaken again with the TissueLyser II for a further minute. Samples were centrifuged and 0.5 ml of 100 % ethanol added to the supernatant before applying samples into the spin columns. DNase treatment in column (Invitrogen PureLink DNase Set) was performed and samples were eluted with RNase-free water. Total RNA was quantified using a Nanodrop Lite Spectrophotometer (Thermo Scientific) and its quality assessed on a 2100 Bioanalyzer (Agilent Technologies, Singapore). High quality RNA (RIN > 8) was used for transcriptome sequencing using Illumina HiSeq 2000 platform to generate 100-nt long paired-end reads. Library preparation and sequencing was performed at Earlham Institute.

### 2.8 RNAseq data analysis or *de novo* transcriptome assembly and annotation

A total of 1.1 billion paired-end reads and a *de novo* transcriptome assembly were generated, and then functionally annotated using the non-redundant (NR) NCBI database, TAIR10, Gene Ontology and Kyoto Encyclopedia of Genes and Genomes (KEGG) (Pruitt et al., 2005; Lamesch et al., 2012; https://www.genome.jp/kegg/, 2020). Quality control analysis was performed on the raw reads using FastQC, followed by Trimmomatic to remove sequencing adapters and trailing low-quality bases. The trinity pipeline was deployed to perform *de novo* transcriptome assembly. All Paired-End (PE) reads were assembled twice using a kmer size of 25 and 30 respectively with normalised reads (minimum coverage = 50, 48 CPUs, 80Gb RAM). Transcripts generated in both assemblies were merged in a single file to maximise the information obtained from both assemblies. CD-HIT EST was subsequently applied to curtail redundancy by merging significantly overlapping contigs with an e-value threshold of 0.95. QUAST v.4.6.3 was used to compare the assembly for the two k-mer-size and the output of CD-HIT EST. Based on these results (**Table S1**) the assembly selected was the one obtained with CD-HIT EST. Abundance estimation of the transcripts obtained was performed using RSEM. Transcripts with very low abundance were removed by filtering by TPM. Quast was employed to display the statistics for the transcriptome filtered by 1 < TPM < 5. Based on these statistics, only transcripts with TPM ≥ 3 were selected in an effort to remove artefacts due to randomly generated transcripts. The selected transcripts were annotated with the Blast2GO PRO functional annotation suite v.4.1.9. (Conesa and Götz, 2008). Blast was performed to find homologous sequences with Blastx-fast against the nr database with a threshold of e-value ≤ 0.001. Blast2GO gene ontology, KEGG pathway and Enzyme annotation were subsequently performed. Blast2GO was also used for Gene ontology enrichment analysis using a matrix of differentially expressed transcripts between different treatments and time points as the test set and the assembled transcriptome as the reference set. The limma R package from Bioconductor (limma, 2018) was used to identify significantly upregulated and downregulated transcripts between different conditions. Transcript counts were imported from RSEM excluding transcripts with low counts (< 20) and a design matrix was created to assign samples to groups. Normalisation was applied using the voom method with quality weights. (Law et al., 2014).

Unsupervised exploratory analysis Principal Component Analysis (PCA) was performed for the resulting dataset. Once a linear model was fit using the design matrix, a series of contrast matrices were created with the aim to find the DE transcripts between ethylene treated samples and control samples, or between different sampling points throughout the storage period. Significantly DE transcripts were considered those with p-value ≤ 0.05 and log fold change (log_2_) ≥ ±1.0.

### 2.9 Statistical analyses and plots

Statistical analyses were conducted using Genstat for Windows 10th Edition (VSN International Ltd, Herts., UK) unless otherwise stated. Analysis of variance (ANOVA) was performed to identify factors or their interactions that significantly affected variance of the data collected. ANOVA was performed on the data specifying a nested treatment structure of a common baseline (observation before post-harvest treatments). The Fisher test, a post-hoc analysis, followed ANOVA. Thus, Fisher least significant difference values (LSD; *P* = 95 %) were calculated from each analysis, for direct comparison between two means from two individual groups (*e.g.* treatment and storage time). Results are significant to p < 0.05, unless otherwise stated. All plots were done using SigmaPlot for Windows SPW13 (Systat Software, Inc., London, UK), unless otherwise stated.

## 3 Results

### 3.1 Exogenous ethylene delayed dormancy and caused a transient increase in respiration rate

**Fig. 2:**
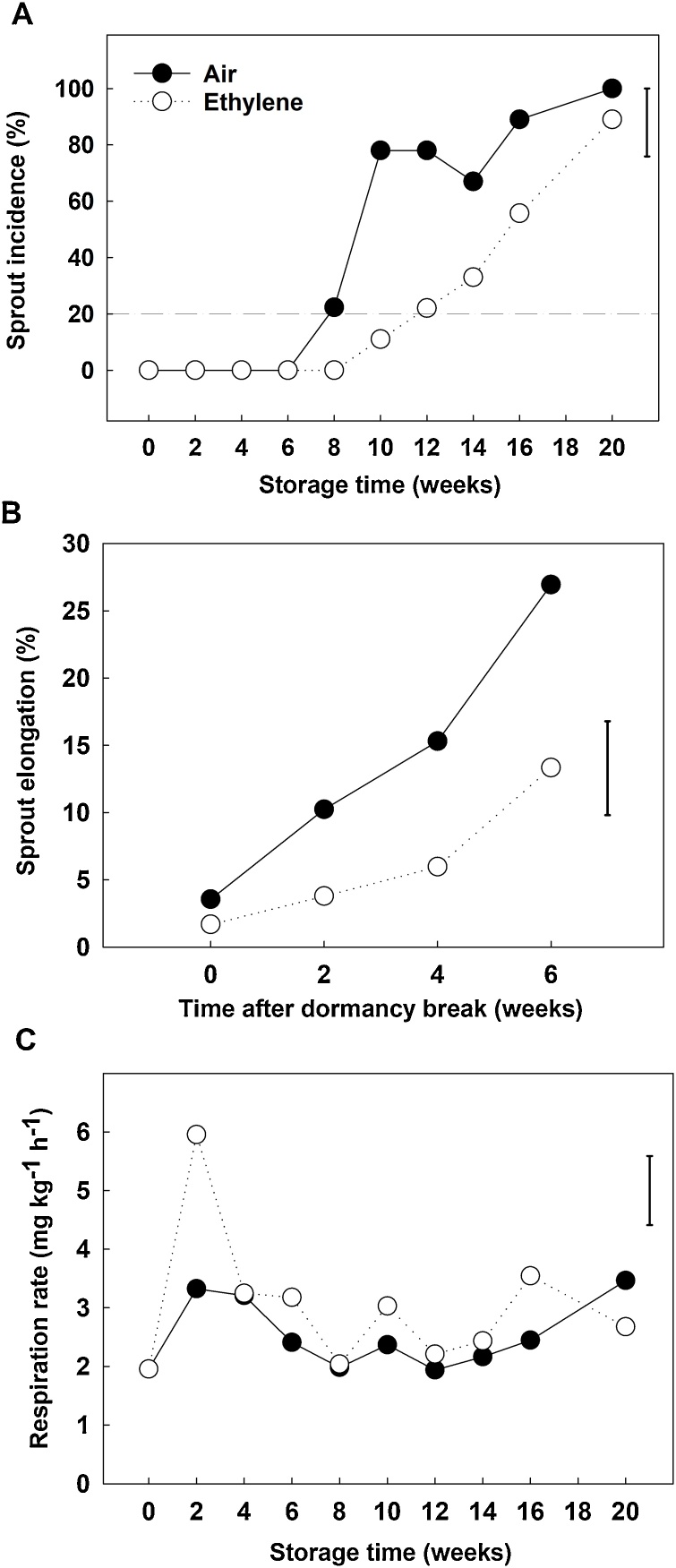
Sprout incidence (presence of sprout elongation as % of total bulbs) (A), where the dash-dot line indicates dormancy break in control onions; bulb sprout elongation (% of total bulb height) (B); and respiration rate as CO_2_ production (mg kg^−1^ h^−1^) (C) for cv. Sherpa bulbs stored at 45 % relative humidity in 2014 (year 2). Bulbs were kept under continuous air (control) or continuous ethylene supplementation (10 μl l^-1^), at 1 °C. Least significance difference (LSD _0.05_) for the interaction treatment x storage time or time after sprouting have been shown.

Ethylene supplementation delayed time to dormancy break by four weeks (Fig. 2A), supporting previous findings (Cools et al., 2011; Ohanenye et al., 2019). In the present study, time to dormancy break was defined as that when the baseplates appeared to be expanded vertically and/or the sprout leaves of at least 20 % of the stored bulbs had resumed elongation. Ethylene supplementation also significantly reduced sprout vigour (Fig. 2B): at 2, 4 and 6 weeks after dormancy break the sprout length of ethylene-treated bulbs was *ca*. half that of the air control (Fig. 2B). No significant differences were found in root incidence between treatments (data not shown). Ethylene treatment resulted in a transient increase (*ca*. 2-fold) in respiration rate at the beginning of the storage period (week 2) (Fig. 2C) which had disappeared by 4 weeks of cold storage, with no significant differences found between treatments thereafter, despite there being a difference in sprout elongation.

### 3.2 Onion *de novo* assembly and annotation

RNA-seq data was obtained from 30 baseplate samples, 12 from the air treatment (control) and 18 from the ethylene treatment. All reads were assembled and annotated (assembly statistics are given in **Table S1**), and a total of 94,840 unigenes were retained after filtering by abundance (TPM ≥ 3), from which 55.2 % had significant BLAST hits, and the remaining 44.8 % (42,439 contigs) had no hits against the NR database (https://www.ncbi.nlm.nih.gov/protein/, 2020). Greater than 30,000 contigs had at least 80 % similarity with known proteins in the NR database. The transcriptome assembly was of high quality and continuity (N50 = 1900 bp), and was therefore used to map individual sample reads for differential expression and downstream analysis. Gene ontology (GO) classifications are presented in **Fig.S2**. The GO terms distribution by biological process was dominated by genes involved in organic substance process (16 %) cellular metabolic process (15 %) and primary metabolic process (15 %), followed by protein nitrogen compound metabolic process (13 %) and biosynthetic process (8 %) (**Fig.S2**A). The GO terms distribution by molecular function (**Fig.S2**B) was dominated by genes involved in organic cyclic compound binding (16 %), heterocyclic compound binding (16 %) and ion binding (14 %); while the most represented genes in the cellular component category were involved in organelle (21 %), intracellular organelle (20 %), cytoplasm (18 %) and membrane component (18 %) (**Fig.S2C**).

### 3.3 A transient effect on global transcription at two weeks of cold storage

Unsupervised exploratory analysis with PCA of the normalised dataset (**Fig.S3**) revealed a clear separation of week 2 samples (for both treatments) from the samples acquired throughout the rest of the storage period (weeks 8, 12, 16 and 20), suggesting that the majority of gene expression changes took place between 2 and 8 weeks regardless of the treatment. However, the excellent separation between treatments at 2 weeks, indiced that there was a strong air *versus* ethylene treatment effect on global gene expression at this time. This separation became much less pronounced at weeks 8 and 12, supporting the idea that a significant part of the continuous ethylene treatment effect was transitory in nature, similar to the respiration transient. These observations are supported by the numbers of differentially expressed genes being highest when contrasting week 2 with any other time point, irrespective of treatment (Table 1).

**Table 1:** Numbers of differentially expressed genes for selected treatment and time contrasts. The number of upregulated, downregulated and total transcripts with Log_2_ >1.0 at adjusted p < 0.05 is given. Air, control treatment (continuous air); Et, ethylene treatment (air supplemented continuously with 10 μl l^−^1 ethylene); w*n*, number of weeks in cold storage at 1 °C; Top, top section of onion baseplate; Bot: bottom section of onion baseplate.

Contrast	Upregulated	Downregulated	Total
Air_w8-Air_w2	1046	1049	2095
Air_w12-Air_w2	813	933	1746
Air_w12-Air_w8	0	0	0
Air_w8_Bot-Air_w8_Top	23	26	49
Et_w12-Et_w2	2834	2869	5703
Et_w8-Et_w2	1825	1991	3816
Et_w12-Et_w8	3	2	5
Et_w16-Et_w8	362	419	781
Et_w16-Et_w2	1411	1710	3121
Et_w16-Et_w12	94	84	178
Et_w20-Et_w8	551	527	1078
Et_w20-Et_w2	1859	1914	3773
Et_w20-Et_w16	1	2	3
Et_w20-Et_w12	33	41	74
Et_w2-Air_w2	592	239	831
Et_w8-Air_w8	267	57	324
Et_w12-Air_w12	244	193	437
Et_w8_Bot-Et_w8_Top	0	0	0

### 3.4 Differential gene expression and GO analysis

**Fig. 3:**
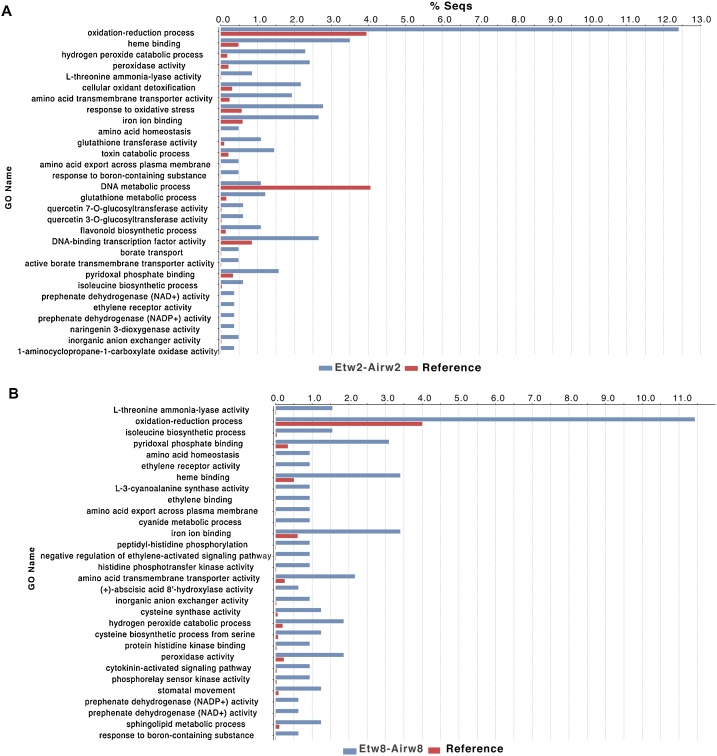
GO enrichment analysis for differentially expressed genes when comparing ethylene-treated *versus* air-treated onion at week 2 (Etw2-Airw2, **A**) and week 8 (Etw8-Airw8, **B**) of cold storage. Bulbs were stored under continuous air (control) or continuous ethylene supplementation (10 μl l^−1^) and RNA was prepared from baseplates. Reference indicates the whole set of unigenes from the *de novo* transcriptome assembly. The x-axis represents the percentage of genes for each GO term for both the reference assembly and for the differentially expressed genes.

A series of expression contrasts were created with the aim of identifying DE transcripts regulating sprout elongation and dormancy break, as well as transcripts responding to exogenous ethylene during storage. The numbers of upregulated/downregulated genes for each contrast are reported in Table 1. When comparing ethylene and air treatments at weeks 2, 8 and 12, there were generally more genes upregulated by ethylene than downregulated (2.2-, 4.7-, and 1.3-fold, respectively). Furthermore, GO enrichment analysis revealed which GO terms were enriched or depleted in the DEs compared to the whole set of unigenes; this analysis was completed for the most interesting contrasts: ethylene-treated *vs.* air-treated samples at weeks 2 (Fig. 3A) and 8 (Fig. 3B). All the GO terms identified were enriched with the exception of “DNA metabolic processes” at week 2, which was a depleted GO term; this suggests DNA metabolism was stable and unaffected by ethylene as far fewer DE trancripts were detected than expected by chance.

### 3.5 KEGG pathway analysis

KEGG pathway analysis for the DE transcripts between ethylene-treated and control samples was performed at week 2 and week 8. The initial timepoint showed a plethora of upregulated pathways in the ethylene treatment compared to control (**Table S2**). These included amino acid metabolism, fructose and mannose metabolism, long chain fatty acid biosynthesis and degradation, nitrogen metabolism and phenylpropanoid biosynthesis. A similar analysis at week 8 (**Table S3**) identified amino acid metabolism, nitrogen metabolism, carotenoid biosynthesis, phenylpropanoid biosynthesis, starch and sucrose metabolism, and glycolysis/glyconeogenesis as pathways containing DE genes. The latter timepoint suggested an upregulation in acetaldehyde and ethanol production, through the upregulation of two transcripts corresponding to an aldehyde dehydrogenase family 2 member B7, mitochondrial-like (EC:1.2.1.3) and an alcohol dehydrogenase gene (EC:1.1.1.1), respectively, indicating that continuous ethylene supplementation regulated anaerobic stress-related genes, even though there was no O_2_ depletion in the ethylene treatment.

To further test the assumptions from the KEGG pathway analysis and GO enrichment analysis, DE trancripts mapping to specific pathways and processes related to ethylene perception and signal transduction, as well as phytohormone pathways that may be involved in dormancy, dormancy break and sprout elongation were explored in a joint analysis of DEGs and hormone levels for each pathway in turn.

### 3.6 Hormone levels changes during dormancy and sprout elongation

#### 3.6.1 ABA and ABA metabolites

**Fig. 4:**
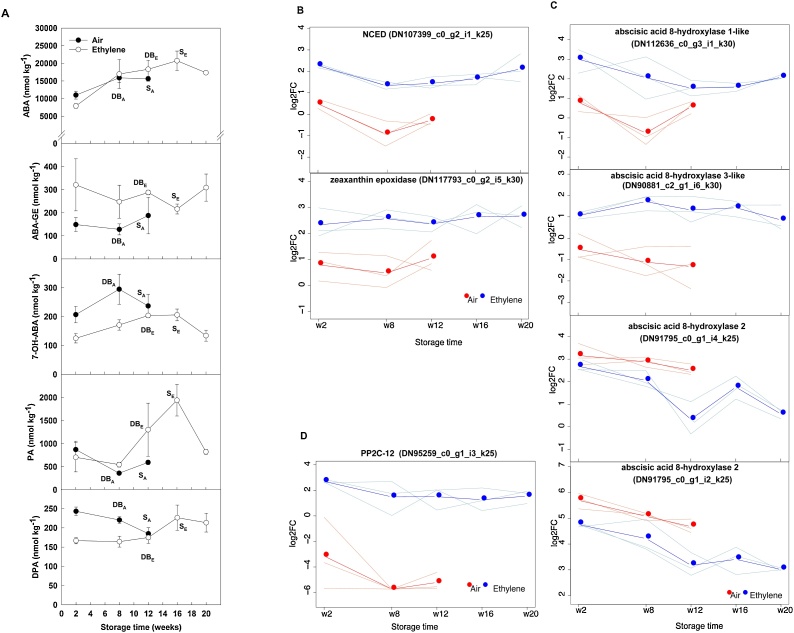
Metabolic profile of ABA and its metabolites, and related transcripts, in onion baseplates. Abscisic acid (ABA) and ABA metabolite concentrations (nmol kg^−1^), per dry weight, are plotted for cv. Sherpa bulb baseplates stored under continuous air (control) or continuous ethylene supplementation (10 μl l^-1^), at 1 °C and 45 % relative humidity (**A**). ABA-GE, ABA-glucose ester; 7−OH-ABA, 7ˊ-hydroxy-ABA; PA, phaseic acid; DPA, dihydrophaseic acid. The physiological stages of onion bulbs during dormancy transition have been indicated next to the corresponding sampling point, by the following acronyms: DB, dormancy break; S, sprout elongation; subscripts A and E indicate air and ethylene treatments, respectively. Each dot in the graph represents the mean value of three replicates (each replicate consisting of three biological replicates). Standard deviations bars are shown for each sampling point. Differentially expressed transcripts related to ABA biosynthesis (**B**); catabolism (**C**); and signalling (**D**), are shown for ethylene-treated (ethylene) and control (air) samples. The y-axis is in log_2_ counts per million (CPM). NCED: *9-cis-epoxycarotenoid dioxygenase;*PP2C-12*: probable phosphatase 2C 12.* Bold lines and dots represent mean values of the three replicates; ligth lines correspond to individual replicates.

When treatments were investigated individually, ethylene supplementation resulted in a significant accumulation (p < 0.05) of ABA in cv. Sherpa baseplates prior to sprout elongation (from 7.9 nmol kg^−1^ at week 2 to 18.3 nmol kg^−1^ at week 12). Contrastingly, in the control treatment, ABA content did not significantly increase from week 2 (11 nmol kg^−1^) to week 12 (peaking at 15 nmol kg^-1^ at week 8); but since the control treatment sprouted earlier, ABA levels could not be compared temporally beyond week 12 (Fig. 4A). If developmentally equivalent stages (dormancy break and sprout elongation) are compared between treatments, there were no significant differences. At the transcriptional level, ethylene supplementation affected the ABA biosynthesis pathway. A transcript annotated as *9-cis-epoxycarotenoid dioxygenase* (NCED - DN107399) and another transcript annotated as *zeathantin epoxidase* (DN117793) were significantly upregulated by ethylene supplementation compared to air at all time points throughout cold storage **(**Fig. 4B). Moreover, a moderate decrease in NCED expression was observed in control onions between 2 and 8 weeks, prior to sprout elongation at week 12, despite the rising ABA levels. At the same time, two isoforms of transcript DN91795, i4 and i2 differing in only one small InDel, annotated as ABA catabolism enzyme *ABA-8ˊ-hydroxylase 2* were downregulated under ethylene supplementation (Fig. 4C); this is consistent with the observed increase in ABA accumulation over time. That said, two different transcripts annotated with the same function, *ABA 8ˊ-hydroxylase 1-like* (DN112636) and *ABA 8ˊ-hydroxylase 3-like* (DN90881), were upregulated (Fig. 4C). PA increased in the ethylene treatment by 4-fold during the transition from dormant (8 weeks) to sprout elongation (16 weeks), however, there was no significant increase in the control treatment which started sprout elongation earlier. Comparing equivalent developmental stages (dormancy break and sprout elongation), PA was higher under the ethylene treatment than in the control. The progressive increase in ABA observed under ethylene could not therefore be explained by a reduction in catabolism to PA, and generally the ABA levels were 10-fold higher than the most abundant metabolite, PA. The catabolites DPA (week 2 and week 8) and 7−OH-ABA (week 8) were significantly higher in control bulbs than ethylene-treated bulbs, but the absolute amounts were approximately 100-fold lower than ABA levels. The higher level of PA observed in the ethylene treatment was consistent with the increased level of transcripts DN90881 and DN112636 encoding putative *ABA 8ˊ-hydroxylase 3-like* and *1-like* enzymes, respectively (Fig. 4C), but was not consistent with the higher level of expression of the two isoforms of transcript DN91795 annotated as *ABA 8ˊ-hydroxylase 2* enzyme which were lower under ethylene (Fig. 4C).

Ethylene supplementation also affected the ABA signalling pathway (Fig. 4D), for example, a transcript annotated as *probable phosphatase 2C 12* (*PP2C-12*) was consistently upregulated (*ca*. by 6 log_2_ count per million [CPM]) throughout the storage period under exogenous ethylene, and interestingly, its expression significantly decreased (p < 0.05) prior to dormancy break in control bulbs.

#### 3.6.2 Cytokinins

**Fig. 5:**
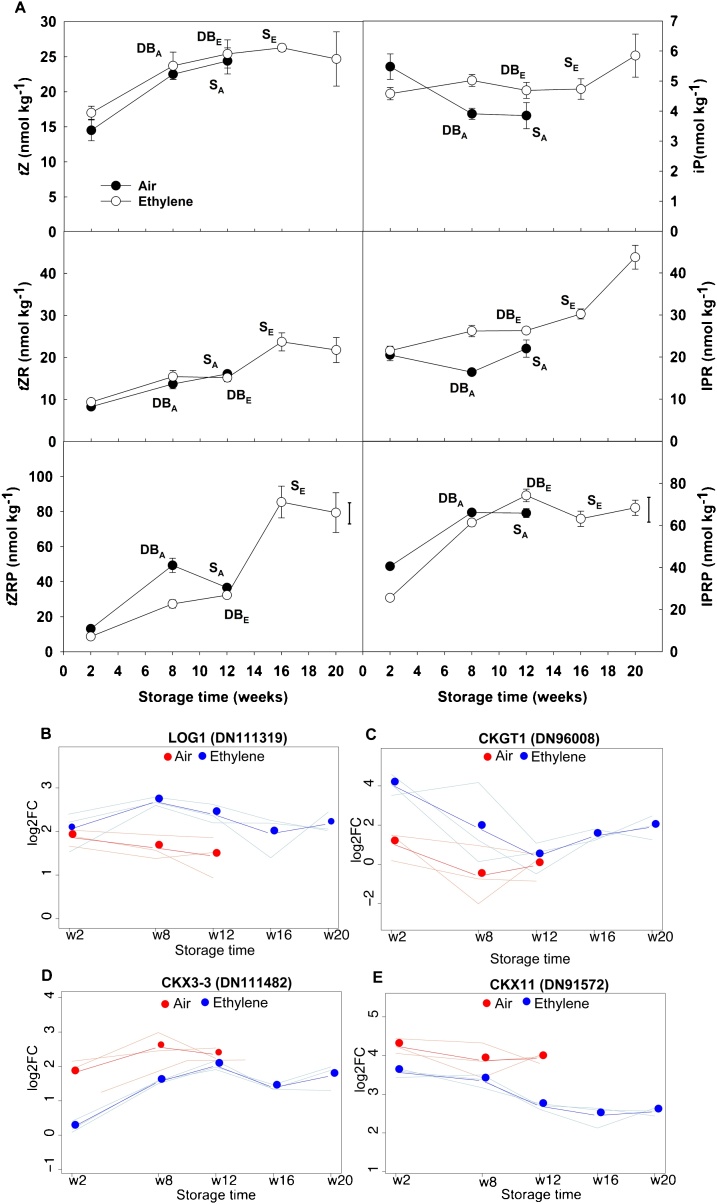
Cytokinins metabolic and transcriptomic profile in onion baseplates. Cytokinins content (nmol kg^−1^) per dry weight, are shown for cv. Sherpa bulb baseplates stored under continuous air (control) or continuous ethylene supplementation (10 μl l^-1^), at 1 °C and 45 % relative humidity (**A**). *t*Z, *trans*-zeatin; *t*ZR, *trans*-zeatin riboside; *t*ZRP, *trans*-zeatin riboside-5ˊ-monophosphate; iP, N^6^-(Δ^2^-isopentenyl)adenine; IPR, isopentenyl-adenosine; IPRP, IPR-5ˊ-monophosphate. The physiological stages of onion bulbs during dormancy transition have been indicated next to the corresponding sampling point, by the following acronyms: DB, dormancy break; S, sprout elongation; subscripts A and E represent air and ethylene treatments, respectively. Each dot in the graph represents the mean value of three replicates (each replicate consisting of three biological replicates). Standard deviations bars are shown for each sampling point. Least significance difference (LSD_0.05_) bars for the significant interaction treatment × storage time are also shown. Differential expression of transcripts is shown for CK biosynthesis gene *cytokinin riboside 5-monophosphate phosphoribohydrolase*-*LOG* (LOG) (**B**) and for CK catabolism genes: *cytokinin-O-glucosyltranferase* (CKGT1) (**C**), *cytokinin dehydrogenase 3-like* (CKX3-3) (**D**) and *cytokinin dehydrogenase 11-like* (CKX11) (*E*). The y-axis is in log_2_ CPM. Bold lines and dots represent mean values of the three replicates; ligth lines correspond to individual replicates.

**Fig. 6:**
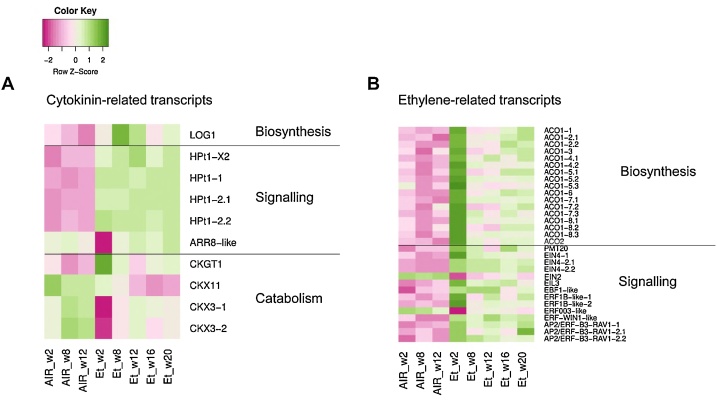
Heatmap describing the expression profile of differentially expressed transcripts (DEs) mapped to cytokinin (**A**) and ethylene (**B**) biosynthesis, signalling and catabolism pathways based on the *de novo* onion assembly. DEs were obtained from all the comparisons possible between continuous ethylene treated (10 μl l^−1^) and control (continuous air) onion bulbs stored at 1 °C and 45 % relative humidity. Et, ethylene supplementation ; ACO, *1-aminocyclopropane-1-carboxylate oxidase*; EIN4, *ETHYLENE-INSENSITIVE4-like*; EIN2, *ETHYLENE-INSENSITIVE2-like*; EIL3, *ETHYLENE-INSENSITIVE3-like3*; EBF1, *ETHYLENE-INSENTITIVE3 (EIN3)-binding F-box 1-like*; ERF1B-like, *ethylene-responsive transcription factor 1B-like*; ERF003-like, *ethylene-responsive transcription factor ERF003-like*; ERF-WIN1-like, *ethylene-responsive transcription factor WIN1-like*; AP2/ERF-B3-RAV1-like, *AP2/ERF and B3 domain-containing transcription factor RAV1-like*; LOG1, *cytokinin riboside 5-monophosphate phosphoribohydrolase LOG1*; HPt1-X2, *histidine-containing phosphotransfer 1-like isoform X2*; HPt1, *histidine-containing phosphotransfer 1-like*; ARR8-like, *two-component response regulator ARR8-like*; CKGT1, *cytokinin-O-glucosyltransferase 1*; CKX11, *cytokinin dehydrogenase 11-like*; CKX3, *cytokinin dehydrogenase 3-like*. For the colour key, pink means downregulation and green represents upregulation of the transcripts represented; the darker the colour, the more differentially expressed the transcripts are. Data have been standardised (mean = 0) by row (row Z-score); values indicate standard deviation from the mean (For interpretation of the references to colour in this figure legend, the reader is referred to the web version of this article).

Several cytokinins (CKs) broadly showed increases in content in baseplates over the course of storage in the presence and absence of added ethylene, most notably the bioactive compound *trans-*zeatin (*t*Z) and its precursors *t*ZR and *t*ZRP. The first member of the cytokinin biosynthetic pathway, isopentenyl adenosine 5ˊ-monophosphate, also increased. However, ethylene had no significant effect on CK content during dormancy transition except for *t*ZRP where ethylene-treated bulbs at dormancy break stage had 1.5-fold lower *t*ZRP concentration than control bulbs at the same development stage (Fig. 5A). The RNAseq analysis showed that ethylene supplementation increased expression of a putative CK biosynthesis gene (LOG gene- Fig. 5B) and reduced the expression of cytokinin oxidase/dehydrogenases (CKX) in the catabolic pathway (Fig. 5C-E). Specifically, a transcript for a key step in CK biosynthesis, *cytokinin riboside 5ˊ-monophosphate phosphoribohydrolase LOG* (DN111319), was upregulated (*ca*. 2-fold) in ethylene from week 8 to week 12 compared to air controls; in contrast, three CKX transcripts (CKX3−1 [DN111482], CKX3−2 [DN111484] and CKX11 [DN91572]) (Fig. 6A) were significantly downregulated in ethylene-treated baseplates compared to control throughout storage. That said, these two groups of transcripts (Fig. 5D and Fig. 5E) had opposite trends during dormancy break and sprout elongation progression: increasing and decreasing for CKX3 and CKX11, respectively. Moreover, *cytokinin-O-glucosyltransferase 1*, which is involved in the deactivation of *t*Z and dihydrozeatin by O-glucosylation, was upregulated at the beginning of the storage period (3.0 log_2_CPM at week 2). However, none of these transcriptional changes correlated with changes in levels of active CKs (*t*Z and iP - Fig. 5A) or inactive CKs, (*t*ZR and *t*ZRP - significantly accumulated by 2- and 4-fold at sprout elongation stage in ethylene-treated bulbs when compared to dormancy break stage) under ethylene supplementation. For week 8, when control onions were undergoing dormancy break and showed initial expansion of their baseplate, a spatial distribution study was carried out: baseplates where cut in half horizontally, and top and bottom sections compared. Ethylene supplementation prevented *t*Z accumulation in the top section of the baseplate (19.4 nmol kg^−1^), compared to control (32.8 nmol kg^−1^) (**Fig.S4**).

The heat map in Fig. 6A depicts how the CK signalling pathway was also altered by ethylene supplementation. Four isoforms of *histidine-containing phosphotransfer 1-like* (*HPt1*) genes (DN100608), immediate downstream targets of cytokinin receptors, were consistently upregulated compared with expression in control bulbs.

### 3.7 Continuous ethylene supplementation upregulates the ethylene biosynthesis and signalling pathway in stored onions

**Fig. 7:**
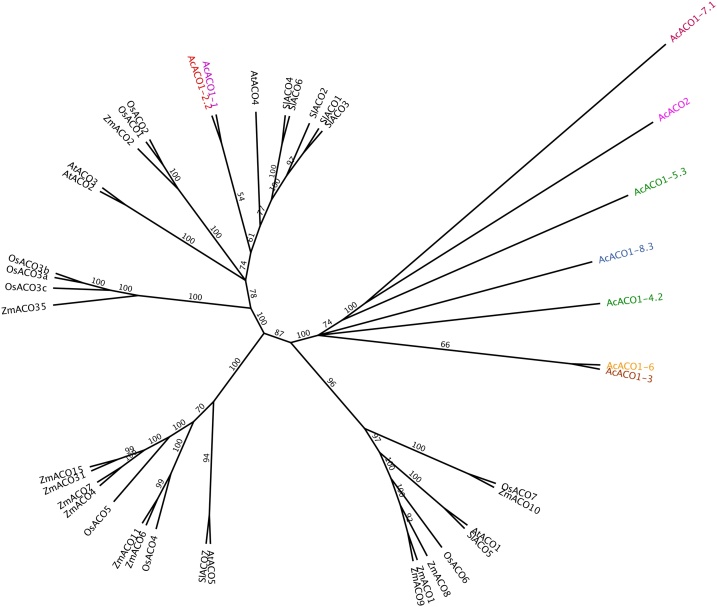
Phylogenetic tree of 1-aminocyclopropane-1-carboxylase oxidase (ACO) protein sequences of cv. Sherpa (Ac) and related protein sequences of Arabidopsis (At), maize (Zm), tomato (Sl) and rice (Zm), based on Houben and Van de Poel (2019). The analysis was conducted in Geneious® (version 9.1.7) using the Neighbor-Joining tree building method, the Jukes-Cantor genetic distance model and with 1000 bootstraps. The numbers indicate bootstrap values.

The ethylene biosynthesis pathway was upregulated in the baseplates of ethylene-treated bulbs (Fig. 6B). A group of eight unigenes encoding for *1-aminocyclopropane-1-carboxylase oxidase 1* (*ACO1*) (DN96798, DN108132, DN124890, DN122774−1, DN122774−2, DN115845, DN110565, DN105069), a key enzyme in ethylene synthesis, and one *ACO2* unigene (DN103877) were identified. A phylogenetic analysis showed that only two *ACO1* unigenes (DN96798 and DN108132 [isoform 1]) were likely to be true ACO1 orthologues (Fig. 7); these candidates were chosen as being closely related to Type I ACO protein sequences with highly conserved domains in Arabidopsis and some monocots such as maize, rice and tomato (Houben and Van de Poel, 2019). Overall, the biggest differences in DE transcripts annotated as ACO were observed at the beginning of the storage (2 weeks) (log_2_ CPM ∼ 2–3); after which the expression sharply decreased prior to dormancy break under ethylene, before increasing again during sprout elongation (Fig. 6B). However, transcripts identified as EIN4 homologs, members of the ethylene receptor family, were significantly upregulated in ethylene-treated bulbs (log_2_ CPM ∼ 2–4 compared with control), and retained similar levels of upregulation throughout storage (Fig. 6B).

An effect of continuous ethylene supplementation on other ethylene signalling components was also observed (Fig. 6B). An *ethylene-insensitive 2-like* (*EIN2*) isoform (log_2_ CPM ∼1.6), which was initially downregulated under ethylene, gradually increased throughout storage reaching similar values to control bulbs at sprouting stage. By contrast, two isoforms annotated as the transcription factor *ethylene-insensitive 3-like 3* (*EIL3*) and a transcript identified as *ethylene-responsive transcription factors 1B-like* (*ERFB1-like*) were upregulated at week 2 (mean log_2_ = 1.5 and 3.1, respectively) and subsequently dropped to the same levels as the control. Moreover, a transcript annotated as *EIN3-binding F-box 1-like* (*EBF1-like*) was upregulated in ethylene-treated onions during the first 12 weeks of storage.

## 4 Discussion

The effect of ethylene on dormancy release is contradictory. In seed germination, ethylene stimulates germination by overcoming the inhibitory effects of ABA pathways (Corbineau et al., 2014), whereas ethylene has also been proposed to inhibit growth by triggering ABA biosynthesis in tomato and cleavers (*Galium aparine*) following application of auxin (Hansen and Grossmann, 2000). Ethylene exposure has been shown to reduce growth in Arabidopsis (Dubois et al., 2018) and to inhibit elongation of young leaves in bulbous plants (Kamerbeek and De Munk, 1976). Abdel-Rahman and Isenberg (1974) showed higher levels of endogenous ethylene production prior to sprout outgrowth, but in contrast, the application of continuous ethylene to onion bulbs is known to delay dormancy break and sprout elongation (Buffler, 2009; Cools et al., 2011; Ohanenye et al., 2019). Cools et al. (2011) investigated the differentially expressed genes between dormant (just after curing) and sprouted onions (after 29 weeks of cold storage); they found that the reduction in sprout elongation of ethylene-treated bulbs was concomitant with the upregulation of *ACO* and *EIN3* transcripts. However, the mechanisms of dormancy break transition in onion bulbs under continuous ethylene supplementation during cold storage were not fully elucidated.

### 4.1 Climacteric-like response to ethylene – respiration and ethylene pathways

This study supports previous observations (Cools et al., 2011; Ohanenye et al., 2019) that ethylene supplementation both delays dormancy break and slows down sprout vigour during postharvest cold storage of onion bulbs. In addition, exogenous ethylene resulted in a climacteric-like response in stored onion where a transient increase in respiration rate (Fig. 2C) was observed. A similar pattern in respiration rate was found by Kamerbeek and Verlind (1972). In their work, iris bulbs stored at 30 °C were subjected to exogenous ethylene ranging from >0.05 to 3 μl l^−1^; a peak in CO_2_ production was observed at 3–4 days of storage after which the respiration rate decreased gradually regardless of the ethylene treatment. In our study, the climactric-like respiration response was also acompained by a significant upregulation of transcripts for *ACO*, a key and limiting step in ethylene biosynthesis (Dubois et al., 2019) (Fig. 6B). However, the *ACO1* upregulation observed in week 2 decreased thereafter compared to control. One explanation for these temporal profiles of ethylene-related genes could be that constant ethylene exposure in onion bulbs leads to a feedback downregulation of the biosynthetic genes.

Additionally, several DE transcripts were found that are orthologous to those that encode proteins involved in the perception of ethylene and the subsequent signal transduction pathways. There was a consistent upregulation of *EIN4*, one of the five receptors found in Arabidopsis to be responsible for ethylene perception: *ETR1*, *ERS1*, *ETR2*, *ERS2* and *EIN4* (Chen et al., 2005). Activation of these receptors triggers a signalling cascade (*EIN2* to *EIN3/EIL* to *ERF*) leading to ethylene-induced responses (Chen et al., 2005; Chang et al., 2013; Dolgikh et al., 2019). When ethylene was applied to Arabidopsis seedlings there was no significant change in *EIN4* transcript abundance following either a 3-day continuous ethylene (1−10 μl l^−1^) application (Hua et al., 1998) or a 6-hour 1 μl l^−1^ ethylene dose (Shakeel et al., 2015), although an increase in the protein level was reported for the latter study.

We also found two DE transcripts orthologous to members of the *EIN3/EIL* Arabidopsis gene family (*EIL* = *EIN3-like*) which contains six transcription factors (*EIN3* and *EIL1−5*); within this family *EIN3*, *EIL1* and *EIL2* are responsible for inducing ethylene response factors (ERFs), and *EIN3* and *EIL3* are the most widely and highly expressed (Dolgikh et al., 2019). The first of these DE genes, annotated as *EIL3,* was consistently upregulated under continuous ethylene supplementation compared to control bulbs and is involved in sulphur deficiency response in Arabidopsis (Dolgikh et al., 2019), whereas *EIN2* was downregulated (Fig. 6B). *EIN3* can also positively regulate *ACO* (Chang et al., 2013), thus it is conceivable that *EIL3* upregulation could lead to increase *ACO*, as found in ethylene-treated baseplates (Fig. 6B), and increase ethylene synthesis since the specific roles of genes within the onion *EIN3/EIL* gene family are unknown and may not be conserved. A similar up-regulation of a tentatively annotated *EIN3* transcript (*i.e.* related to *EIL3* from this study) was observed by Cools et al. (2011) in bulk onion samples (storage sheaths and baseplate) when bulbs were treated with ethylene during 29 weeks of cold storage. However, in the same study both *EIN3* and ethylene receptor-related transcripts were down-regulated after a short (24 h) ethylene treatment, suggesting a more complex temporal regulation which was not addressed in the current study (here the first time point was 2 weeks after the beginning of continuous ethylene supplementation).

Constrastingly, the DE transcript *EIN2,* a positive regulator in ethylene signalling was downregulated in ethylene-treated bulbs; and there was a simultaneous upregulation of the negative ethylene signalling regulator *EBF1-like.* Taken together, it can be seen as an attempt from the bulbs to regulate (negative feedback) the effect of the continuous ethylene on sprout elongation. The subsequent regulation of their expression **(**Fig. 6B**)** upon dormancy break, to reach a similar level to the control could be interpreted as an attenuated sensitivity or adaptation to ethylene; a hypothesis that has been suggested elsewhere (Cools et al., 2011).

We also found that the only ethylene receptor that was differentially expressed was *EIN4*. It was consistently upregulated throughout storage in ethylene-treated bulbs and mirrored the expression profile of *EIL3*. It is known that among the *EIN3* targets there are many that are negative regulators such as the receptors *ETR2* and *ERS1/2*, yet not *EIN4* (Chang et al., 2013). This could be understood as an increase in the ethylene sensitivity of ethylene-treated bulbs. However, this would be counteracted by the decrease in the upregulation of *ACO1* after 2 weeks of ethylene exposure.

Additionally, exogenous ethylene altered the expression of *EBF1*, another ethylene signalling-related transcript. *EBF1* targets *EIN3* for degradation and acts as an ethylene-responsive negative regulator (Chang et al., 2013), so the upregulation of the negative regulator *EBF1-like* and the simultaneous downregulation of the positive regulator *EIN2* observed in ethylene-treated onions suggests there are mechanisms that may allow adaptation to prolonged high ethylene exposure that could potentially explain the transiatory increase in both ethylene biosynthesis transcripts and respiration rate. Moreover, the significant upregulation of multiple *ERFs* (*ERF1B-like-1/2*, *ERF003-like* and *AP2/ERF B3-RAV1*) indicates that the activation of the ethylene signalling pathway likely extends downstream to a cascade of events.

### 4.2 Ethylene-induced delay in dormancy break and sprout elongation is not mediated by ABA in cold storage

In the continuous exogenous ethylene treatment, ABA accumulated during the transition from dormant to sprout elongation stage, but the increase in control bulbs was smaller and not significant, possibly because of the shorter time to dormancy break. The expression of a transcript encoding 9-*cis*-epoxycarotenoid dioxygenase (*NCED*), a rate limiting enzyme in the biosynthesis of ABA (Thompson et al., 2000), was strongly upregulated in ethylene compared to control, but showed a tendency to decline in both treatment between 2 and 8 weeks. Since ABA has previously been hypothesized to inhibit sprouting in onion bulbs, and to decline prior to dormancy break (Chope et al., 2012), the current results do not support an ABA-related mechanism for the ethylene-induced delay in sprout elongation, and an ABA independent mechanism must be involved. However, it has to be noted that this is the first time that ABA, and other hormones, have been quantified in onion baseplates, as opposed to others (Cools et al., 2011; Chope et al., 2012) who reported the hormonal profile during cold storage in samples containing both onion baseplates and storage sheaths. This could explain why in our study the level of ABA was considerably higher (overall mean of *ca*. 14,000 nmol kg^−1^ *vs*. 72−100 nmol kg^−1^) to that previously reported.

The observed ABA accumulation could be due to enhanced biosynthesis and/or reduced catabolism. It was found that an accumulation of PA occurred in baseplates prior to sprout elongation, peaking at 16 weeks when the average content was *ca*. 1.9 nmol kg^−1^. It was also found that a transcript related to ABA catabolism, *ABA8*ˊ*-hydroxylase 2,* was down-regulated under ethylene supplementation, but that *ABAacid 8*ˊ*-hydroxylase 3-like* and *1-like* were up-regulated. The increase in PA seen under ethylene treatment would be consistent with an increased ABA 8ˊ-hydroxylase activity, and so with the increased expression of the 3-like and 1-like forms, but enhanced ABA concentration would also tend to lead to high PA accumulation without a change in ABA 8ˊ-hydroxylase activity just because of a higher substrate concentration. The PA itself is unlikely to have a physiological effect because it is present at 10-fold lower concentration than ABA and it has a lower biological activity. In rice (a monocotyledonous species as is onion) there are three reported ABA 8ˊ-hydroxylase genes *OsABA8ox1*, *OsABA8ox2* and *OsABA8ox3.* It is known that *OsABA8ox1* is induced by ethylene under flooding conditions, and this functions to enhance elongation of submerged shoots (Saika et al., 2007). *OsABA8ox2* and *OsABA8ox3* are repressed in seeds during glucose treatments that delay germination and explain ABA accumulation under these conditions (Shu et al., 2009). *OsABA8ox3* was induced by rehydration of drought-treated plants to rapidly reduce ABA levels during recovary (Cai et al., 2014). Therefore, the presence in onion of different ABA 8ˊ-hydroxylase genes with different responses to ethylene treatment is perhaps not surprising given the range of potential functions of this multigene family. A Clustal alignment of the three onion *ABA 8ˊ-hydroxylase* related genes with monocotyledonous (rice) and dicotyledonous (Arabidopsis) sequences gave species-level gene clustering, so little could be inferred from the literature based on conservation of known functions of different *ABA 8ˊ-hydroxylase* gene family members. The conflicting directions of gene expression for the different onion *ABA 8ˊ-hydroxylases* may explain the lack of a significant ethylene treatment effect on ABA accumulation at equivalent time points.

### 4.3 Ethylene treatment decreases cytokinin *t*ZRP

Cytokinins have previously been reported to have a role in dormancy release in other storage organs (Turnbull and Hanke, 1985a, b; Hartmann et al., 2011). In our study, a steady increase in the main bioactive cytokinin, *t*Z, was observed in onion baseplates over storage time, but was independent of ethylene treatment. Chope et al. (2012) also found that cytokinin level (*viz*. *t*ZR) increased in onion bulbs during storage and reached a maximum at the onset of sprouting. The overall transcript evidence indicated ethylene enhanced cytokinin biosynthesis (increased *LOG* expression) and reduced catabolism (suppressed *CKX* expression), but the measurements of CKs in the baseplate did not closely reflect these trends. Instead both control and ethylene-treated tissues showed genereal increases in CK levels during storage. Two reasons can be advanced for the lack of correlation between transcripts and hormone levels. First, the increases in CKs during storage may indicate compounds delivered from outside the baseplate rather than within. In other studies of cytokinin changes during bud growth initiation, rapid cytokinin increases were found, but the biosynthetic genes, in this case isopententyltransfereases (*IPTs*), did not change expression within buds. Instead, substantial increases in *IPT* expression occurred in subtending stem tissue (Young et al., 2014), and could be repressed by exogenous auxin that also suppressed bud growth. The second reason could be that cytokinin pool sizes are under tight feedback control, for example increased CK levels can lead to upregulation of catabolism through increased *CKX* expression (Brugière et al., 2003).

A steep increase in *t*ZRP at the sprout elongation stage of ethylene-treated bulbs (week 16 - Fig. 5A) was found in the present study, and may indicate an ethylene-mediated mechanism that limits the production of active *t*-Z, perhaps *via* the reduced levels of *LOG* over this period (Fig. 5B). The initial step in cytokinin biosynthesis is catalysed by adenosine phosphate-isopentenyltransferase (IPT), whilst the cytochrome P450 mono-oxygenases CYP735A1 and CYP735A2, and the cytokinin nucleoside 5ʹ-monophosphate phosphoribohydrolases (LOG genes) are key enzymes for two subsequent steps in the biosynthesis of active CKs (Hirose et al., 2008). Typically, *t*Z and *t*ZRP (and *t*ZR) are highly correlated (Young et al., 2014), suggesting that they are under similar regulation. As *t*ZRP is a precursor of *t*Z (LOG step) (Hirose et al., 2008), then high *t*ZRP may either (a) lead to high levels of *t*Z when there is non-limiting LOG or (b) indicate a block in further conversion due to low LOG. The transcriptomics data revealed that the expression of one *LOG* gene, which was upregulated during the first 8 weeks of storage under ethylene treatment, subsequently declined by the time sprouting was initiated at week 16. The changes in *LOG* expression may directly affect bioactive CK pool sizes and/or may be the indirect consequence of feedback regulation as discussed above for *CKX* genes.

The more detailed study of phytohormones distribution within the baseplate itself at week 8 of cold storage (dormancy break of control onions: control baseplates swollen compared to those of ethylene-treated bulbs) revealed that baseplate sections responded differently to exogenous ethylene supplementation (**Fig.S4**); resulting in a differential spatial distribution of *t*Z between bottom and top baseplate sections. The *t*Z concentration was significantly higher in the top baseplate section of control onions, compared to the top baseplate of ethylene-tretaed bulbs. It can then be hypothesised that dormancy break may be controlled by partial inhibition of *t*Z under external ethylene supplementation (**Fig.S4**). At the same time, ethylene resulted in an increase of *t*Z in the bottom baseplate section relative to control. This increase in CK content in the bottom section of baseplate could be related to root development (Werner et al., 2001; Werner et al., 2003). However, no differences in rooting between ethylene-treated and control bulbs were observed. This is in contrast with Cools et al. (2011), who found that continuous ethylene reduced the incidence of rooting when onion bulbs were assessed after a longer storage period (25 weeks), when the rooting process may have been more advanced.

## 5 Conclusions

A *de novo* transcriptome was developed to understand the mechanisms of ethylene supplementation in influencing dormancy transition. The transcriptomics analysis revealed that ethylene supplementation affected several different components of the ethylene, ABA and CK pathways including biosynthesis, catabolism, perception, and signalling. Our work has shown that onion bulbs subjected to continuous ethylene supplementation experience a transient peak in respiration rate and an upregulatuon of *ACO1*, which are reminiscent of a climacteric-like response. This is surprising given that onions are low ethylene producers. The fact that the ethylene-related transcripts did not have a constant response to continuous ethylene supplementation supports the hypothesis that onion bulbs show an adaptative response to the continuous exposure. The concentration of ABA and its catabolite phaseic acid increased over storage time under exogenous ethylene supplementation, whilst the the ABA biosynthesis gene *NCED* was upregulated compared to control. The CK precursors, *t*ZRP and IPRP, significantly increased prior to sprout elongation, and therefore could be considered as potential markers of dormancy status.

## Author statement

M. Carmen Alamar designed and performed most of the experimental work, and wrote the article with contributions from all authors. Leon A. Terry, Colin G.N. Turnbull, Fady Mohareb and Andrew J. Thompson conceived the research plans and supervised the work; Rosa Lopez-Cobollo prepared RNA and performed phytohormone profiling with technical support from Mark H. Bennett; Maria Anastasiadi carried out transcriptomic data analysis; Leon A Terry agrees to serve as the author responsible for contact.

## Declaration of Competing Interest

The authors have no conflict of interest that would bias the collec-tion, analysis, reporting or publishing the research in the manuscript
