# Supplementary material for: Transcriptome and phytohormone changes associated with ethylene-induced onion bulb dormancy
Source: Postharvest Biol Technol. 2020 Oct;168:111267. doi: 10.1016/j.postharvbio.2020.111267 (PMC7398043; doi:10.1016/j.postharvbio.2020.111267)
Supplement: Supplementary file 1 [file mmc1.docx]

**Supplementary Figures**

| **A** 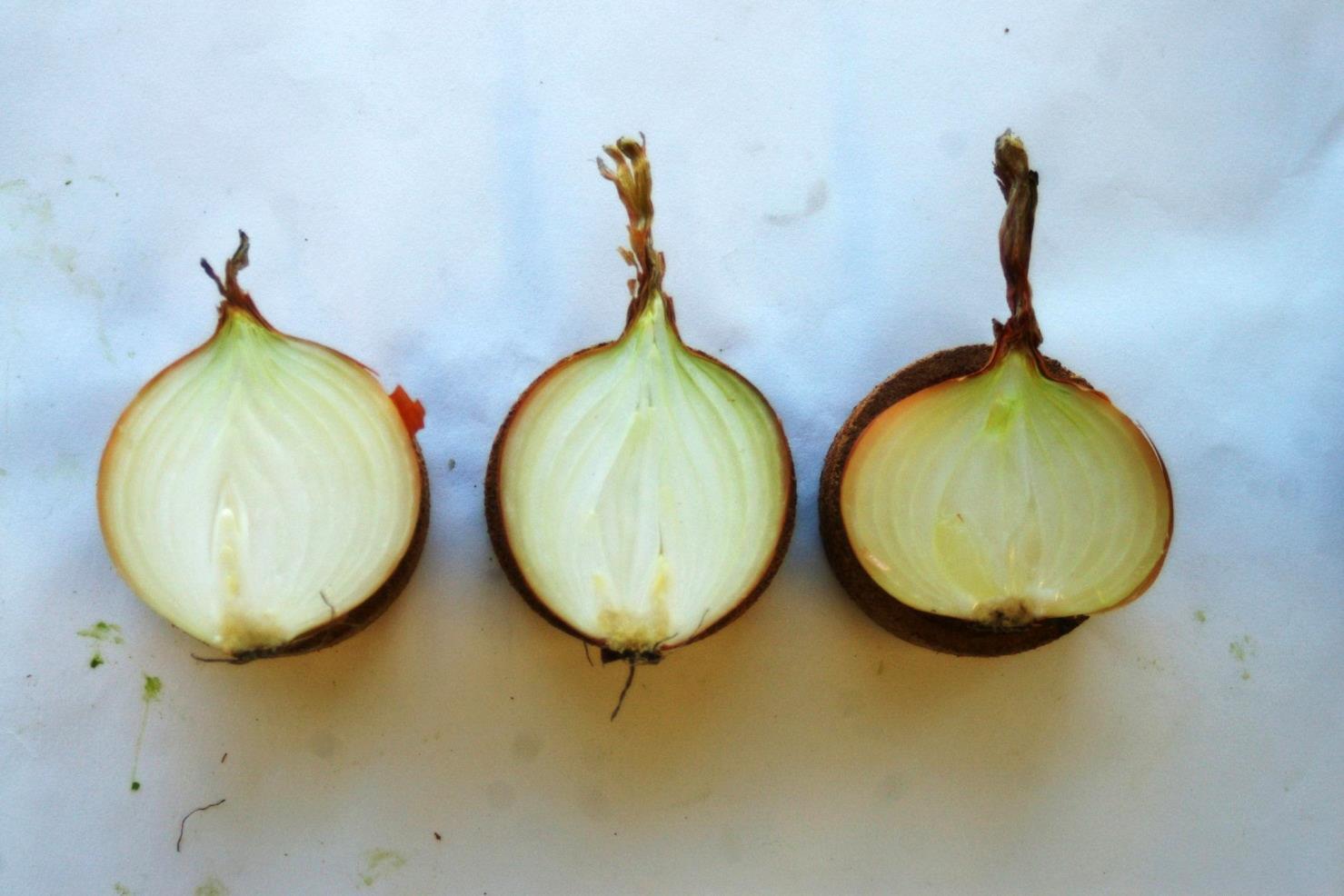 |
| --- |
| **B**  **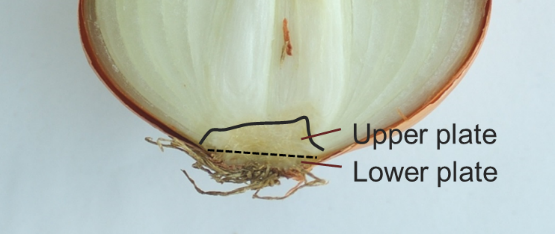** |

**Figure S1.** Pictures showing how onion bulbs were cut transversally for sprout assessment (**A**) - arrows indicate the sprouts that were measured in mm; and how the meristematic tissue/baseplate that excised, for biochemical analysis (**B**).

| 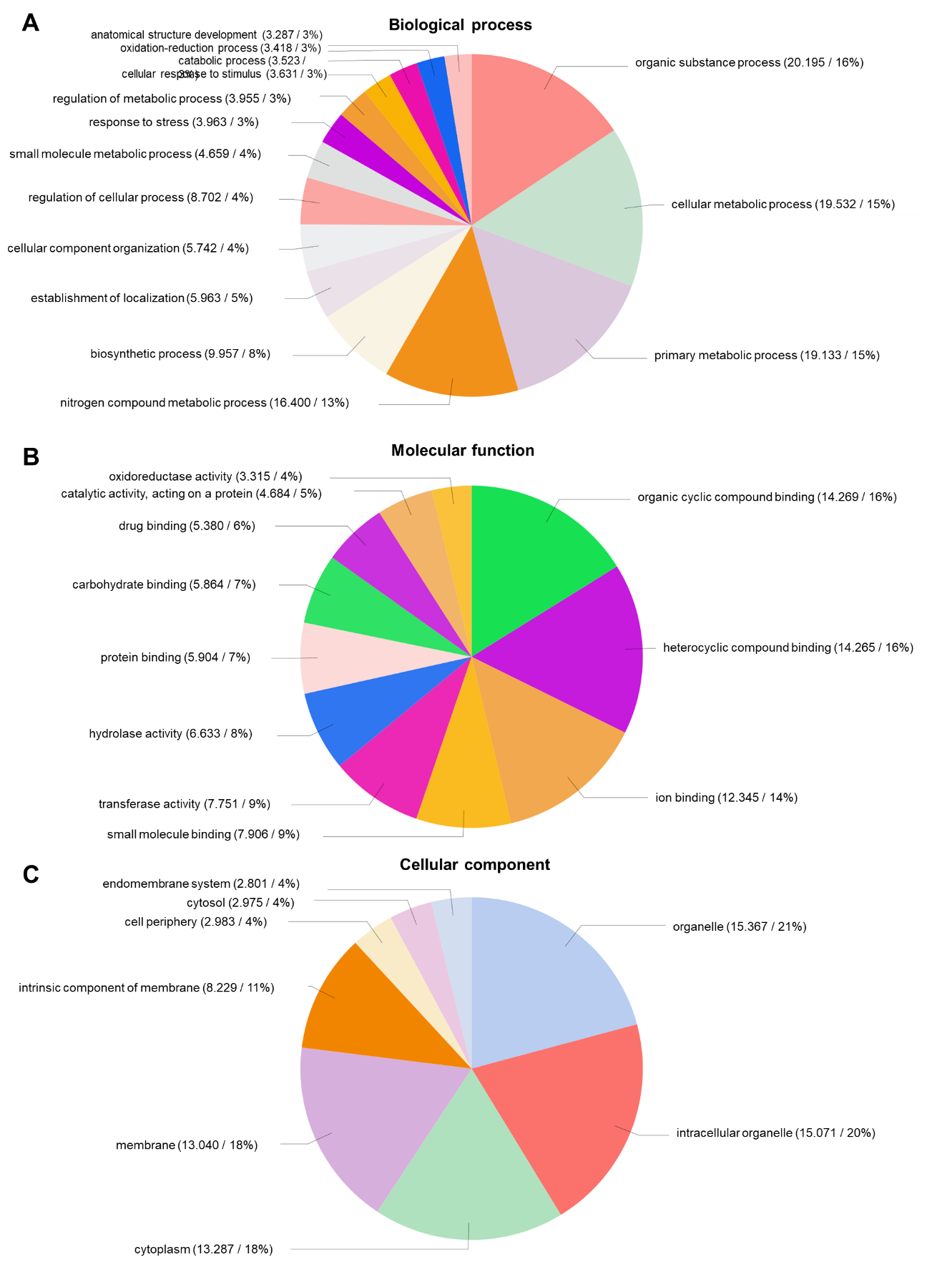 |
| --- |

**Figure S2.** Top 10 highly represented GO terms for biological process (**A**), molecular function (**B**) and cellular component (**C**) in baseplates of cv. Sherpa bulbs kept under continuous air (control) or continuous ethylene supplementation (10 mg kg^-1^) at 1 °C for 12 and 20 weeks, respectively.

| 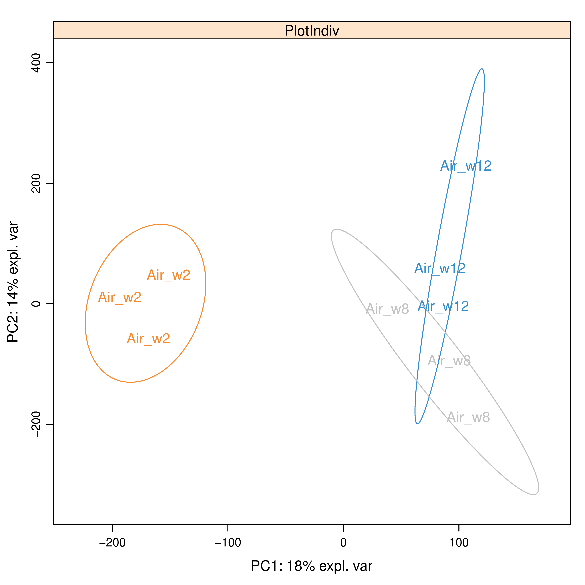 |
| --- |
| 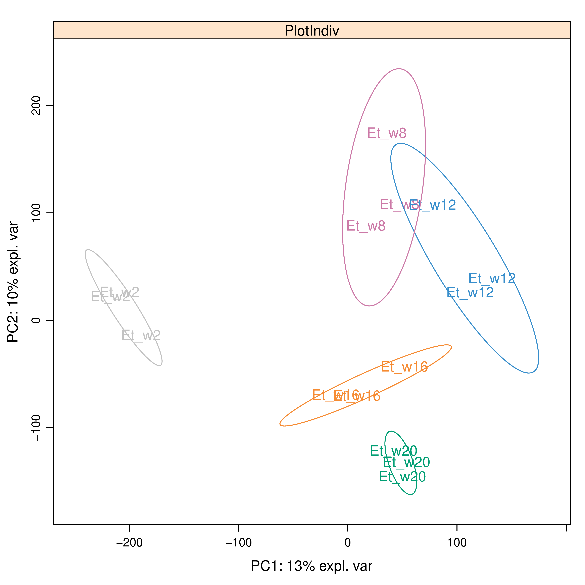 |
| 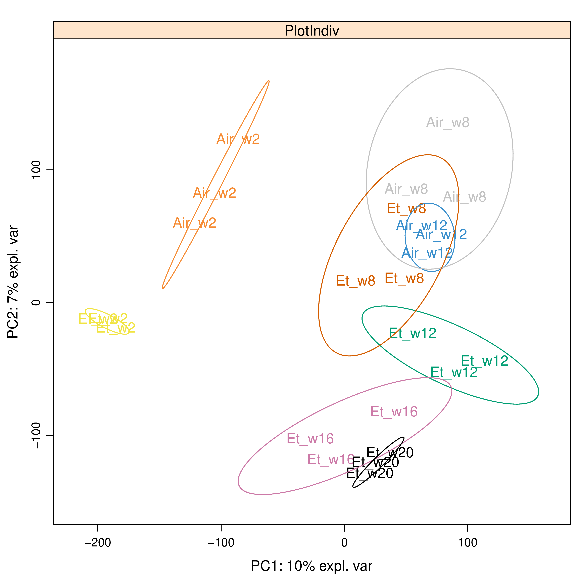 |

**Figure S3.** Unsupervised transcriptomic exploratory analysis. PCA plot for RNA-seq data from baseplate samples in control (**A**), ethylene treatment (**B**) and control and ethylene treatment combined (**C**). Air, control treatment (continuous air); Et, ethylene treatment (air supplemented continuously with 10 mg kg^-1^ ethylene); w*n*, number of weeks in cold storage at 1 °C.

|  |
| --- |

**Figure S4.** Spatial distribution of *trans*-zeatin (*t*Z) in onion baseplate. *t*Z concentration (nmol kg^-1^), per dry weight, in top and bottom bulb baseplate sections from cv. Sherpa stored under continuous air or continuous ethylene supplementation (10 mg kg^-1^), at 1 °C and 45 % relative humidity for 8 weeks. Error bars respresent least significance difference (LSD_0.05_) for the interaction treatment × tissue type (at week 8).

**Tables**

**Table 1.** Numbers of differentially expressed genes for selected treatment and time contrasts. The number of upregulated, downregulated and total transcripts with Log_2_ >1.0 at adjusted *P* < 0.05 is given. Air, control treatment (continuous air); Et, ethylene treatment (air supplemented continuously with 10 mg kg^-^1 ethylene); w*n*, number of weeks in cold storage at 1^°^C.

| **Contrast** | **Upregulated** | **Downregulated** | **Total** |
| --- | --- | --- | --- |
| Air_w8-Air_w2 | 1046 | 1049 | 2095 |
| Air_w12-Air_w2 | 813 | 933 | 1746 |
| Air_w12-Air_w8 | 0 | 0 | 0 |
| Air_w8_Bot-Air_w8_Top | 23 | 26 | 49 |
| Et_w12-Et_w2 | 2834 | 2869 | 5703 |
| Et_w8-Et_w2 | 1825 | 1991 | 3816 |
| Et_w12-Et_w8 | 3 | 2 | 5 |
| Et_w16-Et_w8 | 362 | 419 | 781 |
| Et_w16-Et_w2 | 1411 | 1710 | 3121 |
| Et_w16-Et_w12 | 94 | 84 | 178 |
| Et_w20-Et_w8 | 551 | 527 | 1078 |
| Et_w20-Et_w2 | 1859 | 1914 | 3773 |
| Et_w20-Et_w16 | 1 | 2 | 3 |
| Et_w20-Et_w12 | 33 | 41 | 74 |
| Et_w2-Air_w2 | 592 | 239 | 831 |
| Et_w8-Air_w8 | 267 | 57 | 324 |
| Et_w12-Air_w12 | 244 | 193 | 437 |
| Et_w8_Bot-Et_w8_Top | 0 | 0 | 0 |

**Supplementary Tables**

**Table S1**. Summary of the *de novo* assembly statistics generated with Trinity. Two different assemblies with kmer = 25 and 30 were constructed. The two assemblies were later merged and redundancy was removed with CD-HIT EST.

| **Statistics without reference** | **Trinity_kmer_25** | **Trinity_kmer_30** | **CD-HIT-EST_c95** |
| --- | --- | --- | --- |
| # contigs | 739579 | 706101 | 715497 |
| # contigs (>= 0 bp) | 739579 | 706101 | 715497 |
| # contigs (>= 1000 bp) | 199271 | 223383 | 218916 |
| # contigs (>= 5000 bp) | 7568 | 12770 | 10915 |
| # contigs (>= 10000 bp) | 210 | 430 | 317 |
| # contigs (>= 25000 bp) | 0 | 0 | 0 |
| # contigs (>= 50000 bp) | 0 | 0 | 0 |
| Largest contig | 16456 | 17969 | 17969 |
| Total length | 665869329 | 735716838 | 718765200 |
| Total length (>= 0 bp) | 665869329 | 735716838 | 718765200 |
| Total length (>= 1000 bp) | 434001713 | 525182114 | 501817108 |
| Total length (>= 5000 bp) | 47997807 | 82206446 | 69706027 |
| Total length (>= 10000 bp) | 2550707 | 4915356 | 3678260 |
| Total length (>= 25000 bp) | 0 | 0 | 0 |
| Total length (>= 50000 bp) | 0 | 0 | 0 |
| N50 | 1569 | 1910 | 1809 |
| N75 | 693 | 863 | 817 |
| L50 | 118560 | 110151 | 113465 |
| L75 | 277735 | 252024 | 260112 |
| GC (%) | 36.14 | 35.98 | 36.21 |

**Table S2**. KEGG pathway enrichment analysis for the contrast ethylene *vs*. control in week 2. Samples were whole baseplates of cv. Sherpa onion bulb stored under continuous air (control) or continuous ethylene supplementation (10 mg kg^-1^), at 1 °C and 45 % relative humidity.

| **KEGG path** | **KEGG pathway** | **Upregulated enzymes** | **Downregulated enzymes** | **Total** |
| --- | --- | --- | --- | --- |
| path:map00010 | Glycolysis / Gluconeogenesis | 1 | 1 | 2 |
| path:map00040 | Pentose and glucuronate interconversions | 5 | 0 | 5 |
| path:map00051 | Fructose and mannose metabolism | 2 | 0 | 2 |
| path:map00052 | Galactose metabolism | 0 | 1 | 1 |
| path:map00053 | Ascorbate and aldarate metabolism | 3 | 0 | 3 |
| path:map00062 | Fatty acid elongation | 1 | 0 | 1 |
| path:map00071 | Fatty acid degradation | 2 | 0 | 2 |
| path:map00130 | Ubiquinone and other terpenoid-quinone biosynthesis | 1 | 0 | 1 |
| path:map00140 | Steroid hormone biosynthesis | 1 | 0 | 1 |
| path:map00190 | Oxidative phosphorylation | 0 | 1 | 1 |
| path:map00220 | Arginine biosynthesis | 1 | 1 | 2 |
| path:map00230 | Purine metabolism | 0 | 2 | 3 |
| path:map00250 | Alanine, aspartate and glutamate metabolism | 3 | 0 | 3 |
| path:map00260 | Glycine, serine and threonine metabolism | 1 | 0 | 1 |
| path:map00261 | Monobactam biosynthesis | 0 | 1 | 1 |
| path:map00270 | Cysteine and methionine metabolism | 1 | 0 | 1 |
| path:map00280 | Valine, leucine and isoleucine degradation | 2 | 0 | 2 |
| path:map00290 | Valine, leucine and isoleucine biosynthesis | 1 | 0 | 1 |
| path:map00300 | Lysine biosynthesis | 0 | 1 | 1 |
| path:map00310 | Lysine degradation | 2 | 0 | 2 |
| path:map00330 | Arginine and proline metabolism | 1 | 0 | 1 |
| path:map00340 | Histidine metabolism | 1 | 0 | 1 |
| path:map00360 | Phenylalanine metabolism | 2 | 0 | 2 |
| path:map00362 | Benzoate degradation | 1 | 0 | 1 |
| path:map00380 | Tryptophan metabolism | 2 | 0 | 2 |
| path:map00400 | Phenylalanine, tyrosine and tryptophan biosynthesis | 2 | 0 | 2 |
| path:map00410 | beta-Alanine metabolism | 2 | 0 | 2 |
| path:map00430 | Taurine and hypotaurine metabolism | 1 | 0 | 1 |
| path:map00460 | Cyanoamino acid metabolism | 1 | 0 | 2 |
| path:map00480 | Glutathione metabolism | 2 | 0 | 2 |
| path:map00500 | Starch and sucrose metabolism | 2 | 0 | 4 |
| path:map00520 | Amino sugar and nucleotide sugar metabolism | 2 | 0 | 2 |
| path:map00531 | Glycosaminoglycan degradation | 1 | 0 | 1 |
| path:map00561 | Glycerolipid metabolism | 1 | 1 | 2 |
| path:map00562 | Inositol phosphate metabolism | 1 | 0 | 1 |
| path:map00564 | Glycerophospholipid metabolism | 0 | 2 | 2 |
| path:map00620 | Pyruvate metabolism | 1 | 1 | 2 |
| path:map00625 | Chloroalkane and chloroalkene degradation | 1 | 0 | 1 |
| path:map00627 | Aminobenzoate degradation | 2 | 0 | 2 |
| path:map00630 | Glyoxylate and dicarboxylate metabolism | 1 | 0 | 1 |
| path:map00640 | Propanoate metabolism | 1 | 0 | 1 |
| path:map00650 | Butanoate metabolism | 1 | 0 | 1 |
| path:map00740 | Riboflavin metabolism | 1 | 0 | 1 |
| path:map00901 | Indole alkaloid biosynthesis | 1 | 0 | 1 |
| path:map00906 | Carotenoid biosynthesis | 1 | 0 | 1 |
| path:map00908 | Zeatin biosynthesis | 0 | 1 | 1 |
| path:map00910 | Nitrogen metabolism | 4 | 0 | 4 |
| path:map00920 | Sulfur metabolism | 0 | 1 | 1 |
| path:map00930 | Caprolactam degradation | 1 | 0 | 1 |
| path:map00940 | Phenylpropanoid biosynthesis | 3 | 0 | 4 |
| path:map00941 | Flavonoid biosynthesis | 2 | 0 | 2 |
| path:map00944 | Flavone and flavonol biosynthesis | 1 | 0 | 1 |
| path:map00945 | Stilbenoid, diarylheptanoid and gingerol biosynthesis | 1 | 0 | 1 |

**Table S3**. KEGG pathway enrichment analysis for the contrast ethylene *vs*. control in week 8. Samples were whole baseplates of cv. Sherpa onion bulb stored under continuous air (control) or continuous ethylene supplementation (10 mg kg^-1^), at 1 °C and 45 % relative humidity.

| **KEGG path** | **KEGG pathway** | **Upregulated enzymes** | **Downregulated enzymes** | **Total** |
| --- | --- | --- | --- | --- |
| path:map00010 | Gly°olysis / Gluconeogenesis | 2 | 0 | 2 |
| path:map00040 | Pentose and glucuronate interconversions | 1 | 0 | 1 |
| path:map00053 | Ascorbate and aldarate metabolism | 2 | 0 | 2 |
| path:map00071 | Fatty acid degradation | 2 | 0 | 2 |
| path:map00130 | Ubiquinone and other terpenoid-quinone biosynthesis | 1 | 0 | 1 |
| path:map00140 | Steroid hormone biosynthesis | 1 | 0 | 1 |
| path:map00230 | Purine metabolism | 1 | 0 | 1 |
| path:map00250 | Alanine, aspartate and glutamate metabolism | 4 | 0 | 4 |
| path:map00260 | Glycine, serine and threonine metabolism | 3 | 0 | 3 |
| path:map00270 | Cysteine and methionine metabolism | 2 | 0 | 2 |
| path:map00280 | Valine, leucine and isoleucine degradation | 1 | 0 | 1 |
| path:map00290 | Valine, leucine and isoleucine biosynthesis | 1 | 0 | 1 |
| path:map00310 | Lysine degradation | 2 | 0 | 2 |
| path:map00330 | Arginine and proline metabolism | 1 | 0 | 1 |
| path:map00340 | Histidine metabolism | 1 | 0 | 1 |
| path:map00350 | Tyrosine metabolism | 1 | 0 | 1 |
| path:map00360 | Phenylalanine metabolism | 1 | 0 | 1 |
| path:map00380 | Tryptophan metabolism | 1 | 0 | 1 |
| path:map00400 | Phenylalanine, tyrosine and tryptophan biosynthesis | 1 | 0 | 1 |
| path:map00410 | beta-Alanine metabolism | 1 | 0 | 1 |
| path:map00430 | Taurine and hypotaurine metabolism | 1 | 0 | 1 |
| path:map00460 | Cyanoamino acid metabolism | 2 | 0 | 2 |
| path:map00480 | Glutathione metabolism | 1 | 0 | 1 |
| path:map00500 | Starch and sucrose metabolism | 2 | 0 | 2 |
| path:map00531 | Glycosaminoglycan degradation | 1 | 0 | 1 |
| path:map00561 | Glycerolipid metabolism | 1 | 0 | 1 |
| path:map00562 | Inositol phosphate metabolism | 1 | 0 | 1 |
| path:map00592 | alpha-Linolenic acid metabolism | 1 | 0 | 1 |
| path:map00600 | Sphingolipid metabolism | 0 | 1 | 1 |
| path:map00620 | Pyruvate metabolism | 1 | 0 | 1 |
| path:map00625 | Chloroalkane and chloroalkene degradation | 2 | 0 | 2 |
| path:map00626 | Naphthalene degradation | 1 | 0 | 1 |
| path:map00627 | Aminobenzoate degradation | 0 | 1 | 1 |
| path:map00630 | Glyoxylate and dicarboxylate metabolism | 2 | 0 | 2 |
| path:map00740 | Riboflavin metabolism | 1 | 0 | 1 |
| path:map00860 | Porphyrin and chlorophyll metabolism | 1 | 0 | 1 |
| path:map00901 | Indole alkaloid biosynthesis | 1 | 0 | 1 |
| path:map00903 | Limonene and pinene degradation | 1 | 0 | 1 |
| path:map00906 | Carotenoid biosynthesis | 2 | 0 | 2 |
| path:map00910 | Nitrogen metabolism | 2 | 0 | 2 |
| path:map00920 | Sulfur metabolism | 1 | 0 | 1 |
| path:map00940 | Phenylpropanoid biosynthesis | 3 | 0 | 3 |
| path:map00941 | Flavonoid biosynthesis | 1 | 0 | 1 |
| path:map00944 | Flavone and flavonol biosynthesis | 1 | 0 | 1 |
| path:map00945 | Stilbenoid, diarylheptanoid and gingerol biosynthesis | 1 | 0 | 1 |
